# High-fraction brookite films from amorphous precursors

**DOI:** 10.1038/s41598-017-15364-y

**Published:** 2017-11-09

**Authors:** James E. S. Haggerty, Laura T. Schelhas, Daniil A. Kitchaev, John S. Mangum, Lauren M. Garten, Wenhao Sun, Kevin H. Stone, John D. Perkins, Michael F. Toney, Gerbrand Ceder, David S. Ginley, Brian P. Gorman, Janet Tate

**Affiliations:** 1 Department of Physics, Oregon State University, Corvallis, OR 97331 USA; 2 Applied Energy Programs, SLAC National Accelerator Laboratory, Menlo Park, CA 94025 USA; 3 Department of Materials Science and Engineering, Massachusetts Institute of Technology, Cambridge, MA 02139 USA; 4 Department of Metallurgical and Materials Engineering, Colorado School of Mines, Golden, CO 80401 USA; 5 National Renewable Energy Laboratory, Golden, CO 80401 USA; 6 Materials Science Division, Lawrence Berkeley National Laboratory, Berkeley, CA 94720 USA; 7 Department of Materials Science and Engineering, UC Berkeley, Berkeley, CA 94720 USA; 8 Stanford Synchrotron Radiation Lightsource, SLAC National Accelerator Laboratory, Menlo Park, CA 94025 USA

## Abstract

Structure-specific synthesis processes are of key importance to the growth of polymorphic functional compounds such as TiO_2_, where material properties strongly depend on structure as well as chemistry. The robust growth of the brookite polymorph of TiO_2_, a promising photocatalyst, has been difficult in both powder and thin-film forms due to the disparity of reported synthesis techniques, their highly specific nature, and lack of mechanistic understanding. In this work, we report the growth of high-fraction (~95%) brookite thin films prepared by annealing amorphous titania precursor films deposited by pulsed laser deposition. We characterize the crystallization process, eliminating the previously suggested roles of substrate templating and Na helper ions in driving brookite formation. Instead, we link phase selection directly to film thickness, offering a novel, generalizable route to brookite growth that does not rely on the presence of extraneous elements or particular lattice-matched substrates. In addition to providing a new synthesis route to brookite thin films, our results take a step towards resolving the problem of phase selection in TiO_2_ growth, contributing to the further development of this promising functional material.

## Introduction

**Figure 1:**
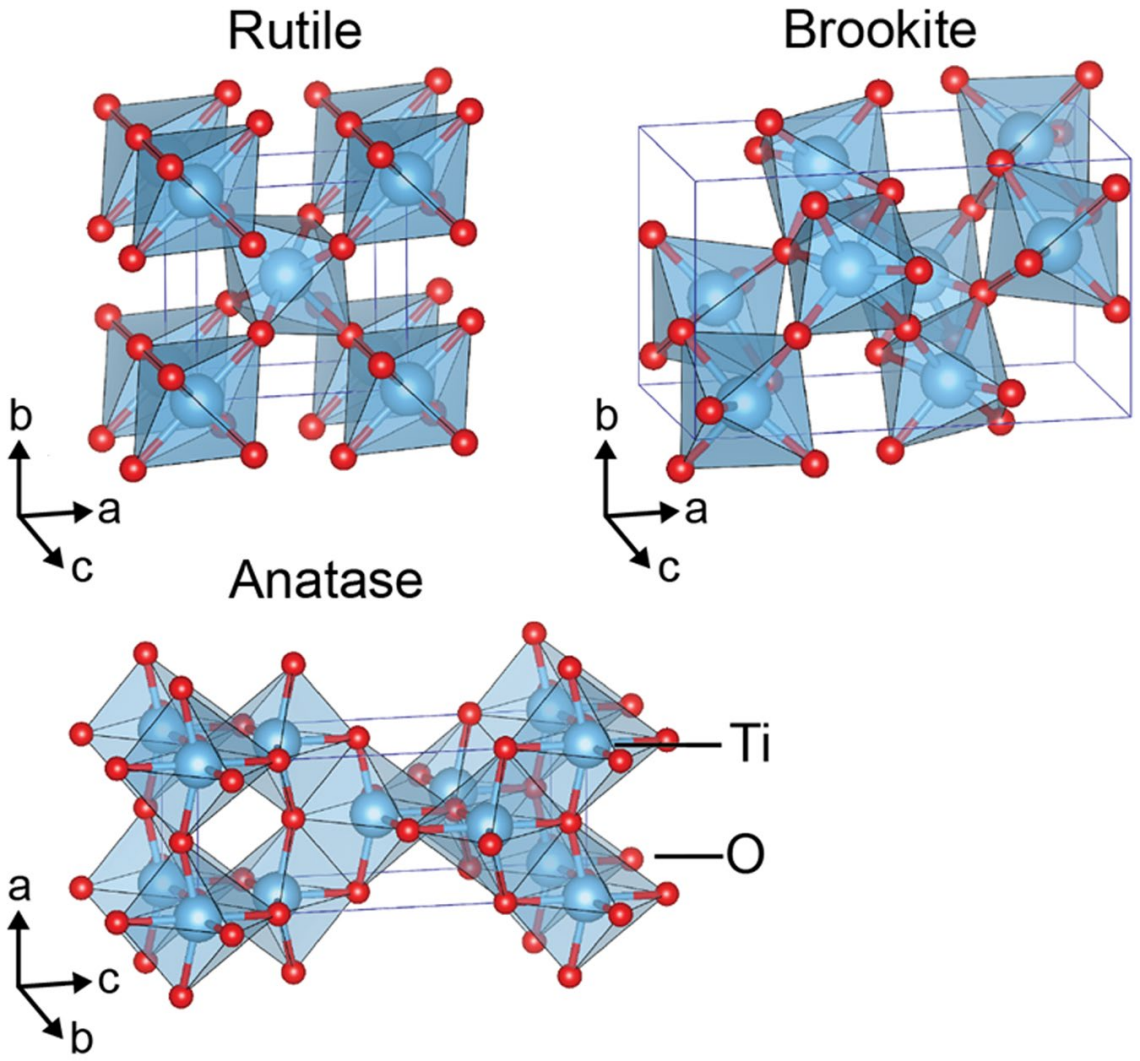
Crystal structures of TiO_2_ rutile (tetragonal, P4_2_/mmm), brookite (orthorhombic, Pbca) and anatase (tetragonal, I4_1_/amd) polymorphs.

TiO_2_ can form in many structures, the most prominent of which are the naturally-occurring rutile, anatase and brookite polymorphs depicted in Fig. 1, although many other synthetically prepared structures have been reported^1,2^. Rutile, the ground state, and anatase are by far the most common and find many applications^3,4^, from paint pigments to transparent conductors and photocatalysts. Brookite is seeing renewed interest^5–7^, with a rutile/brookite mixture serving as effective photocatalysts and outperforming Degussa P25 (an anatase/rutile mixture commonly used to degrade organic pollutants) at degrading both rhodamine B and methyl orange. Furthermore, through surface engineering, brookite can become either oxidative or reductive^8^ depending on the exposed surface ((201) or (210) respectively), adding versatility as a catalyst. However, the synthesis of high-quality brookite has proven much more difficult than that of rutile and anatase, motivating us to study its formation in the form of a functionally-relevant thin film.

Designing a synthesis route to a metastable phase such as brookite requires the knowledge of both equilibrium and non-equilibrium reactions that could occur^9^. Although phase diagrams of the bulk Ti-O system under standard conditions are established, phase selection during synthesis is not merely a matter of bulk equilibrium thermodynamics, as evidenced by the ubiquity of metastable, non-equilibrium synthesis products, which include anatase and brookite. As reported in similar ionic systems, synthesis routes to various stable and metastable phases can be driven by finite-size effects^10^, nucleation kinetics^11,12^, bulk and surface off-stoichiometry^13,14^ and others^15,16^. Table 1 lists several proposed stabilization mechanisms for the common TiO_2_ polymorphs, as well as the conditions driving their transformations. In the case of brookite, it appears that the most efficient reactions leading to the bulk material are crystallization by sol-gel and hydrothermal techniques in the presence of Na or some other alkali ion spectators^17,18^. However, there is no established mechanistic understanding of these synthesis routes. Moving further beyond bulk synthesis, the growth of materials as thin-films opens the door to high-energy synthesis methods such as sputtering and pulsed laser deposition (PLD). Although thin films of brookite have been obtained by vapour deposition on lattice-matched substrates^19–22^ (Table 2), the non-equilibrium growth of high-purity brookite on amorphous substrates has been largely unexplored.

**Table 1:** Formation mechanisms reported for some metastable TiO_2_ polymorphs.

TiO_2_ Polymorph/Transformation	Stabilization/Formation Mechanism
Anatase	Smaller crystal sizes and lower pressure^45^.
Anatase → Rutile	Growth post-nucleation to a large crystal size, reconstructive process^3^
Anatase → Baddeleyite	Small crystallite sizes under high pressures^45^
Anatase → α-PbO_2_	Large nanocrystals to macroscopic single crystals under high pressures^45^
Anatase → Brookite	Controlled size in the tens of nanometers regime and the presence of surfactants^10,46,47^.Twinning on anatase {112} facets^48^.
Brookite	Helper ions such as NaOH^17^, C_2_H_2_O_4_ + polymer^24^, pH during hydrothermal reaction^8^, or Cl^-^ ions^25^ during either solution or hydrothermal synthesis. Intermediate crystallite sizes^10^.

All polymorphs transform to rutile above 600–700 °C.

**Table 2:** Vapour deposition of phase pure brookite reported in the literature.

PVD Method	Substrate	Thickness (nm)
PEALD^19^	(110) YSZ	~80
MAPLE^20^	(100) Si	~50
PECVD^22^	(100) Si	Unknown
MOCVD^21^	(110) YSZ	60

The lack of a clear and consistent mechanistic understanding of brookite growth explains the wide variety of hydrothermal and sol-gel synthesis approaches found in literature^3,4^. Some reports find that high concentrations of NaOH lead to the best brookite samples^23^, while others use a combination of inorganic and organic precursors to produce phase pure brookite^24^, and others report pure brookite based on a delicate balance between Cl^−^ ion concentration, pH, and the necessity of the [Ti(OH)_2_(Cl)_2_(OH_2_)_2_]^0^ salt as a brookite precursor^25^. One common thread between these recipes is the use of some ionic complexing agent (Na^+^, C_2_O_4_^2−^, or Cl^−^) to favor the structural selection of brookite in large quantities. However, vapour deposition processes inspired by the proposed growth mechanisms do not consistently lead to the same results^26^.

One of the reasons for the discrepancies between solution-based and vapour-based results, and the difficulty of synthesizing brookite via the vapour phase, and in particular by physical vapour deposition (PVD), could be the delicate balance of thermodynamic forces favoring each polymorph^10^, which is difficult to transfer precisely between growth techniques. Another possibility is that helper species present during hydrothermal or sol-gel growth (e.g. Na^+^, C_2_O_4_^2−^, Cl^−^) are not typically used during PVD growth, limiting the accessible pathways to the ones without these helper species. As a result, to date synthesis of pure brookite by vapour deposition has relied on highly specific stabilization mechanisms, namely substrate lattice-matching, to produce any thickness of thin-film brookite. The first study to produce brookite via vapour deposition^22^ relied on plasma-enhanced chemical vapour deposition (PECVD) with titanium tetra-isopropoxide (a common precursor used in TiO_2_ hydrothermal growth) vapour on (100)-oriented silicon substrates. Other works^19,21^ have reported lattice-matched brookite growth on (110) yttria-stabilized zirconia (YSZ) by plasma-enhanced atomic layer deposition (PEALD) and metal-organic chemical vapour deposition (MOCVD), reporting an in-plane orientation of [001]_brookite_//[001]_YSZ_ and an out-of-plane orientation of [120]_brookite_//[110]_YSZ_. In one case, a brookite nanorod film was reported^20^ grown by matrix-assisted pulsed laser evaporation (MAPLE), using a target made from a suspension of solution-synthesized brookite nanorods frozen in liquid nitrogen, onto (100) Si substrates. Furthermore, of the brookite formation studies on amorphous glass substrates^27,28^, the highest brookite fraction reported to date is 45 ± 15%^27^ with the remaining film composed of rutile and anatase. Thus, despite the relative abundance of synthesis routes yielding brookite from solution, there remains no established mechanism reported for the formation of high-fraction brookite on general, non-lattice-matched or amorphous substrates.

In this work, we report a simple, substrate agnostic synthesis route that produces a high fraction of the brookite phase in a thin film by PLD of amorphous TiO_2_ and subsequent annealing. We characterize the growth process and resulting films through 2D synchrotron X-ray diffraction (XRD), 2D micro-Raman spectroscopy, high resolution transmission electron microscopy (HRTEM), energy dispersive X-ray spectroscopy (EDS) and first-principles calculations to confirm the presence of brookite and discuss the possible mechanisms leading to its formation. We are able to produce thin films with more than 90% brookite, document the interplay of brookite with the more common rutile and anatase polymorphs, and eliminate a number of reaction mechanisms previously proposed on the basis of sol-gel and hydrothermal growth results. Specifically, we investigate the proposed role of Na in promoting brookite growth as it migrates into the growing film from the substrate^26,29,30^, and demonstrate that contrary to previous reports^17,18^, brookite growth does not depend on the presence of Na. Instead, the nucleation and growth of brookite appears to rely on processing and structural properties such as film thickness.

## Results

### Theory

Based on a variety of hydrothermal and sol-gel brookite synthesis recipes, a common claim is that the presence of sodium during TiO_2_ growth promotes the formation of the brookite phase^17,18^. To investigate the thermodynamic feasibility of this claim, we evaluate the thermodynamics of sodium incorporation into various known TiO_2_ phases. This analysis follows by analogy to the recently reported role of alkali ions in the formation of MnO_2_ polymorphs through the stabilization of off-stoichiometric intermediate products^13^. To identify possible off-stoichiometric intermediates, we examine the formation of partially sodiated Na_x_TiO_2+y_ compounds, with Na^+^ incorporated into the Ti-O matrices of each phase (rutile, brookite, and anatase, as well as the bronze, layered, and postspinel structures) which are known to form in the Na-TiO_2_ space^31^, so that the Na_x_TiO_2+y_ phases may template the growth of a particular Ti-O framework. To enumerate the Na_x_TiO_2+y_ structures that may play a role in such a mechanism, we consider Na^+^ incorporation either through the partial reduction of Ti^4+^ to Ti^3+^, or the formation of Ti^4+^ vacancies (equivalent to the incorporation of Na_2_O). Note, we do not consider the incorporation of purely interstitial Na_2_O as none of the TiO_2_ polymorphs contain interstitial sites large enough for such defects. We then evaluate the stability of these structures by comparing their energies to that of all known phases in the Na_x_TiO_2_ and Na_2y_TiO_2+y_ spaces, regardless of Ti-O framework.

**Figure 2:**
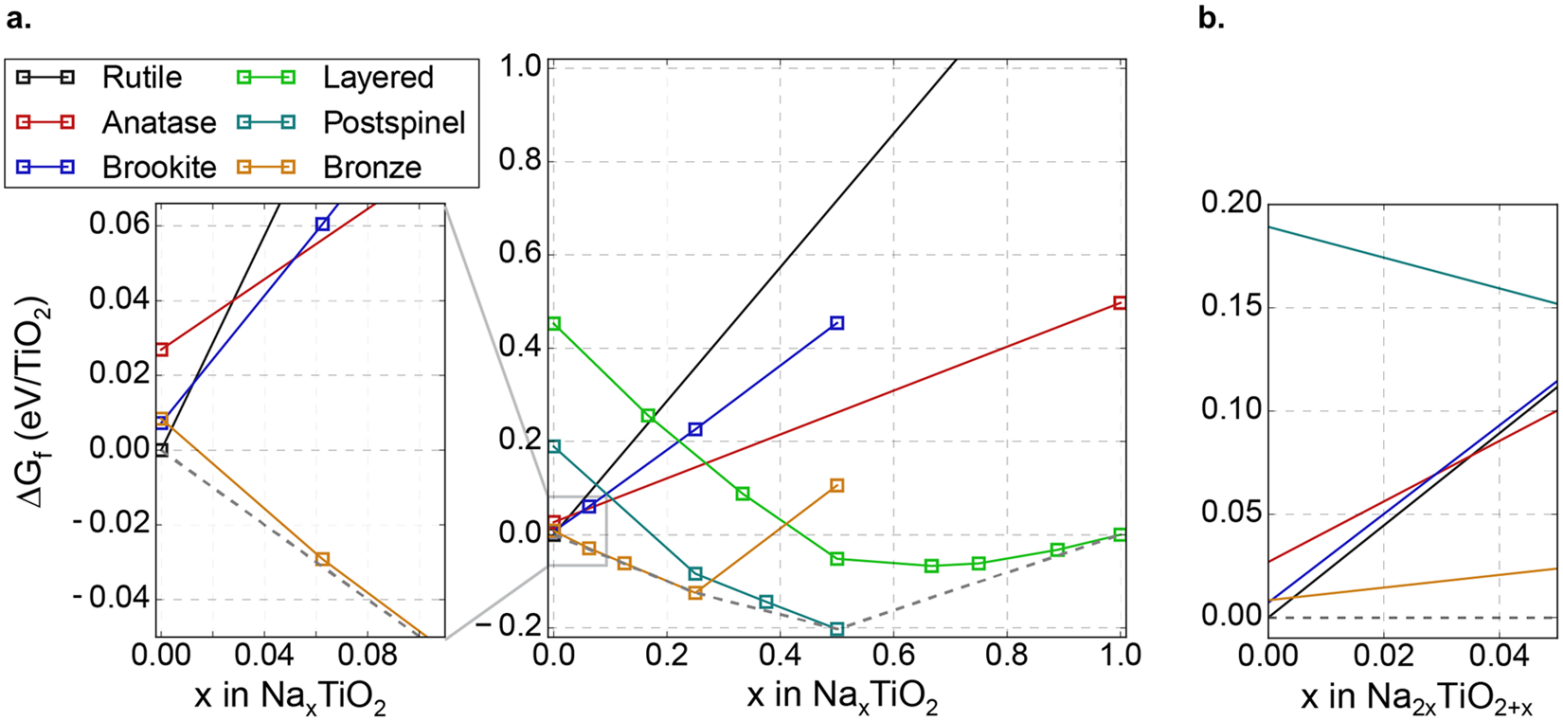
Computed low-temperature free energies of (**a**) Na_x_TiO_2_ and (**b**) Na_2y_TiO_2+y_ compounds, with Ti-O frameworks constrained to those of known TiO_2_ phases, as a representative sample of structure selection in the Na_x_TiO_2+y_ chemical space. The dotted lines denote the global thermodynamic equilibrium in each composition space.

The low-temperature, low-pressure thermodynamics of Na_x_TiO_2+y_ compounds, shown as heats of formation in Fig. 2(a) and (b), do not support the previously hypothesized role of sodium in brookite formation, at least via the formation of a bulk-sodiated intermediate. In the case of Na^+^ intercalation alongside Ti^4+/3+^ reduction, the stabilized phases are TiO_2_-bronze at low sodium content, post-spinel at intermediate sodiation, and layered-TiO_2_ at high sodiation. These results are consistent with both reported crystal structures in this space^31^, and the observation that small levels of sodiation appear to favor the TiO_2_-bronze phase^26^. Similarly, Na_2_O incorporation is most favorable in the TiO_2_-bronze phase, and generally leads to the well-known Na_2_Ti_3_O_7_ titanate structure, which is not topotactically related to any of the TiO_2_ polymorphs we consider. While these results are obtained for zero-temperature conditions, the large difference in Na-defect energies seen in Fig. 2 would make the entropic stabilization of sodiated brookite over the competing phases highly unlikely. Of course, it is possible that the role of sodium in previous experiments was not to influence the relative stability of TiO_2_-phases through bulk incorporation – for example, sodium may selectively influence the surface energies of specific TiO_2_ polymorphs, thereby affecting their relative nucleation kinetics, analogous to the role of Mg^2+^ ions in influencing CaCO_3_ polymorphism^12^. While we expect that the impact of surface sodium on the relative surface energies of the TiO_2_ polymorphs would be limited to 0.1 J/m^2^,^13^ a rigorous analysis of sodiated TiO_2_ interfaces would be necessary to ascertain the exact behavior of interfacial Na, and falls outside the scope of this work. Nonetheless, our analysis suggests that sodium may not be essential to brookite formation, motivating our further experimental study of brookite growth in both sodium-containing and sodium-free media.

### Observation of brookite in films on different substrates

Amorphous TiO_2_ films deposited on a variety of amorphous substrates, and annealed as described in the methods section, consistently produce crystalline films with varying quantities of brookite and anatase, and sometimes rutile, regardless of whether the substrate is sodium-free (pure fused silica (a-SiO_2_) or Si with thermally-grown oxide) or sodium-containing glass (Corning Eagle XG (EXG) with low Na content, or soda-lime-silicate (SLS) with high Na content). Na clearly plays no role in the brookite formation on the Na-free substrates. We detail below the identification of various titania phases in a typical film deposited on low-Na EXG glass. Notably, we find no evidence of Na migration into that film. If sodium incorporation were indeed favorable, sodium migration from the substrate would have produced sodiated TiO_2_^26,29,30^. We find the fraction of brookite produced in this process is controlled by the thickness of the film and the time and temperature of the anneal, rather than the substrate and the presence of Na^+^.

**Figure 3:**
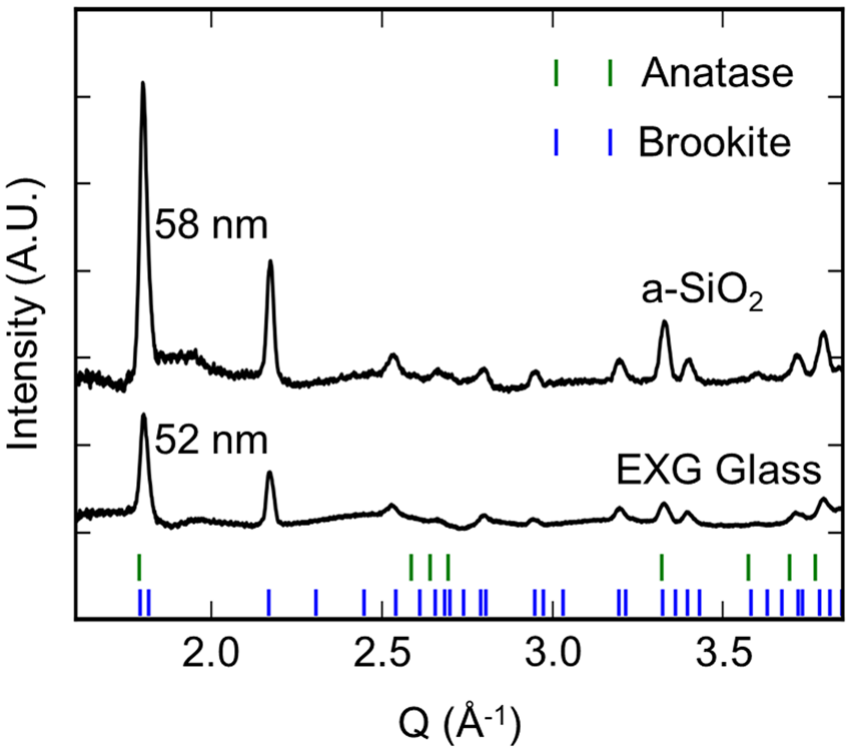
Room temperature XRD patterns of TiO_2_ films grown on a-SiO_2_ and EXG substrates annealed following a protocol similar to that shown in Fig. 4. Optical images of these films appear in Fig. S3. The brookite and anatase phases appear in similar proportions in films on both substrates.

Figure 3 shows background-subtracted, integrated 2D XRD spectra of TiO_2_ films, 58 nm and 52 nm thick, on Na-free fused SiO_2_ and Na-containing EXG glass substrates respectively, obtained after annealing. The strong brookite signature in films on both Na-containing and Na-free substrates is evident from the XRD peak at Q = 2.166 Å^−1^. This result shows that films of the same thickness that are annealed under the same conditions can produce similar mixed phase brookite and anatase films regardless of the presence of Na in the substrate. These are examples of high-fraction brookite films (>70% brookite), where the fraction is quantified from the optical and Raman maps as discussed below.

**Figure 4:**
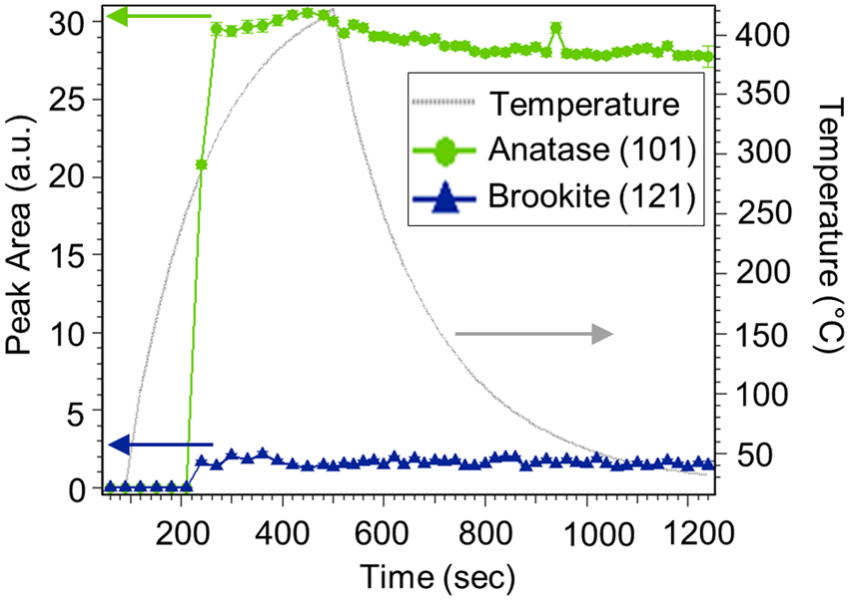
The integrated peak areas for brookite (121) [blue] and anatase (101) [green] on a 65-nm film, grown on EXG, as a function of time and temperature during the anneal indicated by the gray line. Both polymorphs crystallize at ~290 °C.

To explore the annealing process and progression of the crystalline phases, we use *in-situ* XRD during annealing. Figure 4 shows the *in-situ* XRD evidence from a 65-nm TiO_2_ (measured by HRTEM) film on amorphous EXG glass annealed in N_2_ according to the annealing profile shown in gray. Brookite is tracked by the (121) peak at Q = 2.166 Å^−1^ which provides a clear signature because it is strong and isolated from the other polymorph peaks. Anatase is tracked by its (101) peak at Q = 1.787 Å^−1^, which is very strong but close to a moderately strong brookite peak. This particular film has a relatively low fraction of brookite (<20%) which makes the anatase identification unambiguous in XRD (an example 2D XRD spectrum can be seen in Fig. S1). Here we observe the crystallization of brookite and anatase TiO_2_ phases at ~290 °C. When the experiment is repeated, we consistently observe that the anatase and brookite polymorphs form concurrently. The polymorphs quickly achieve their final steady-state phase fractions, as evidenced by the lack of change in peak area after 200 secs. In contrast to crystallization from solution, which is often dominated by one polymorph at a time^32^, the concurrent formation of two separate polymorphs here suggests independent nucleation events, likely at different locations of the substrate. There is minimal transformation of the two phases after formation. Gibbs’ phase rule suggests that there can only be one polymorph at equilibrium in the film at a time, so the persistence of a two-polymorph thin film suggests slow coarsening kinetics, which is likely due to limited transport kinetics in thin films.

**Figure 5:**
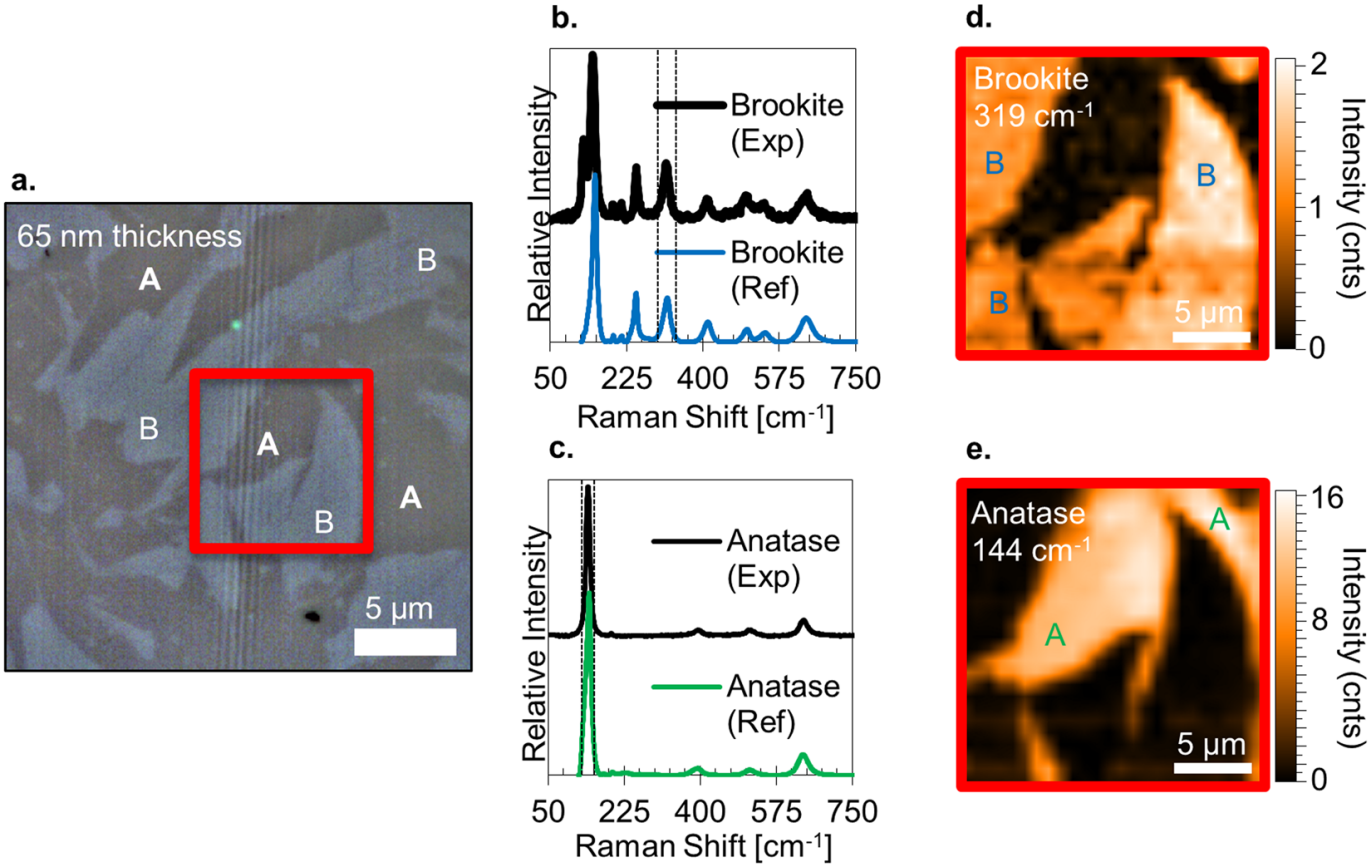
(**a**) Optical image (100x magnification) of a 65-nm TiO_2_ film on EXG glass. Regions marked B yield the Raman spectrum for brookite in (**b**), while those marked A yield the anatase spectrum in (**c**). The 2D Raman maps at 319 cm^−1^ (**d**) and 144 cm^−1^ (**e**) show that the color variations in the optical image correlate with a particular polymorph. The brookite:anatase ratio in this film is 50:50 and no rutile is observed. The dotted lines in (**b**) and (**c**) indicate the wavenumber range over which the Raman intensity maps in (**d**) and (**e**), respectively, are acquired.

**Figure 6:**
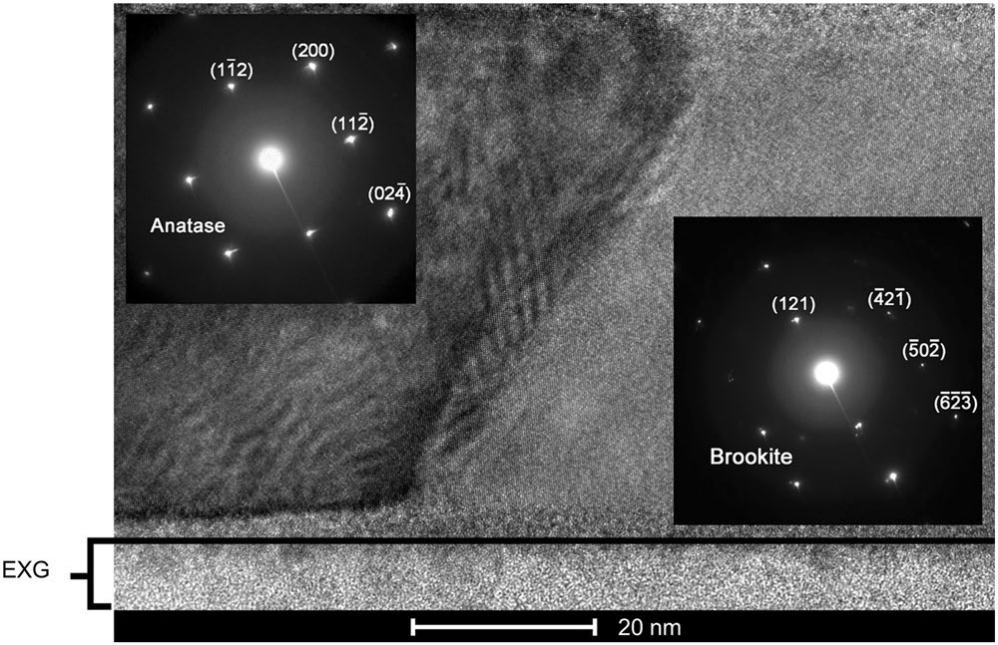
HRTEM image of a 50-nm TiO_2_ film on EXG glass annealed following a protocol similar to that shown in Fig. 4. The selected area electron diffraction patterns in the insets indicate that the grain on the left is anatase and on the right, is brookite. The S-shaped grain boundary is approximately 20 nm wide.

Figure 5 shows optical and Raman images of the mixed-phase brookite/anatase film in Fig. 6. Figure 5a is an optical image, with clear contrast evident on the micron length scale. The lighter regions, labeled B, are characterized by the Raman spectrum in Fig. 5b which matches the brookite spectrum in the RRUFF database (RRUFFID R050591)^33^. The darker regions, labeled A, yield the Raman spectrum in Fig. 5c, which matches anatase (RRUFFID R070582)^33^. With the Raman shift tuned to 319 cm^−1^ (brookite) and 144 cm^−1^ (primarily anatase), the laser is scanned over the region marked by the red square in Fig. 5a, and the 2D maps in Fig. 5d and e, respectively, are obtained. The shapes evident in the optical image, which presumably result from refractive index contrast (*n*_rutile_ = 2.72, *n*_brookite_ = 2.64, *n*_anatase_ = 2.53)^34^, are replicated in the Raman maps, giving an unambiguous polymorph identification. The optical contrast therefore provides a quick and convenient “first-cut” polymorph identification method, which can then be confirmed with Raman or electron microscopy. From the 2D Raman maps or the optical images, it is easy to convert the A and B areas to polymorph fractions, which for this film is 50% brookite to 50% anatase. Associating the area with a volume phase fraction assumes the surface structures permeate the film.

Cross-sectional TEM analysis in Fig. 6 shows that the grains do indeed occupy the entire film thickness and can be considered columnar on a micron-scale. Figure 6 also provides further phase confirmation by the electron diffraction shown in the insets. The TEM image shows an anatase-brookite grain boundary, which changes curvature from the top of the film to the bottom. This S-shape extends over a 20-nm range perpendicular to the boundary. Due to a balance of forces indicated by the point of inflection, boundary mobility and further grain growth are significantly reduced, again suggesting that phase stability is kinetically limited^35^. The *in-situ* XRD studies in Fig. 4 also suggest a rapid settling into a particular morphological configuration after a rapid nucleation, shown by the plateau in intensity after crystallization. Further experiments are needed to understand the details of the nucleation and growth of the polymorphs and the nature of the phase boundary.

**Figure 7:**
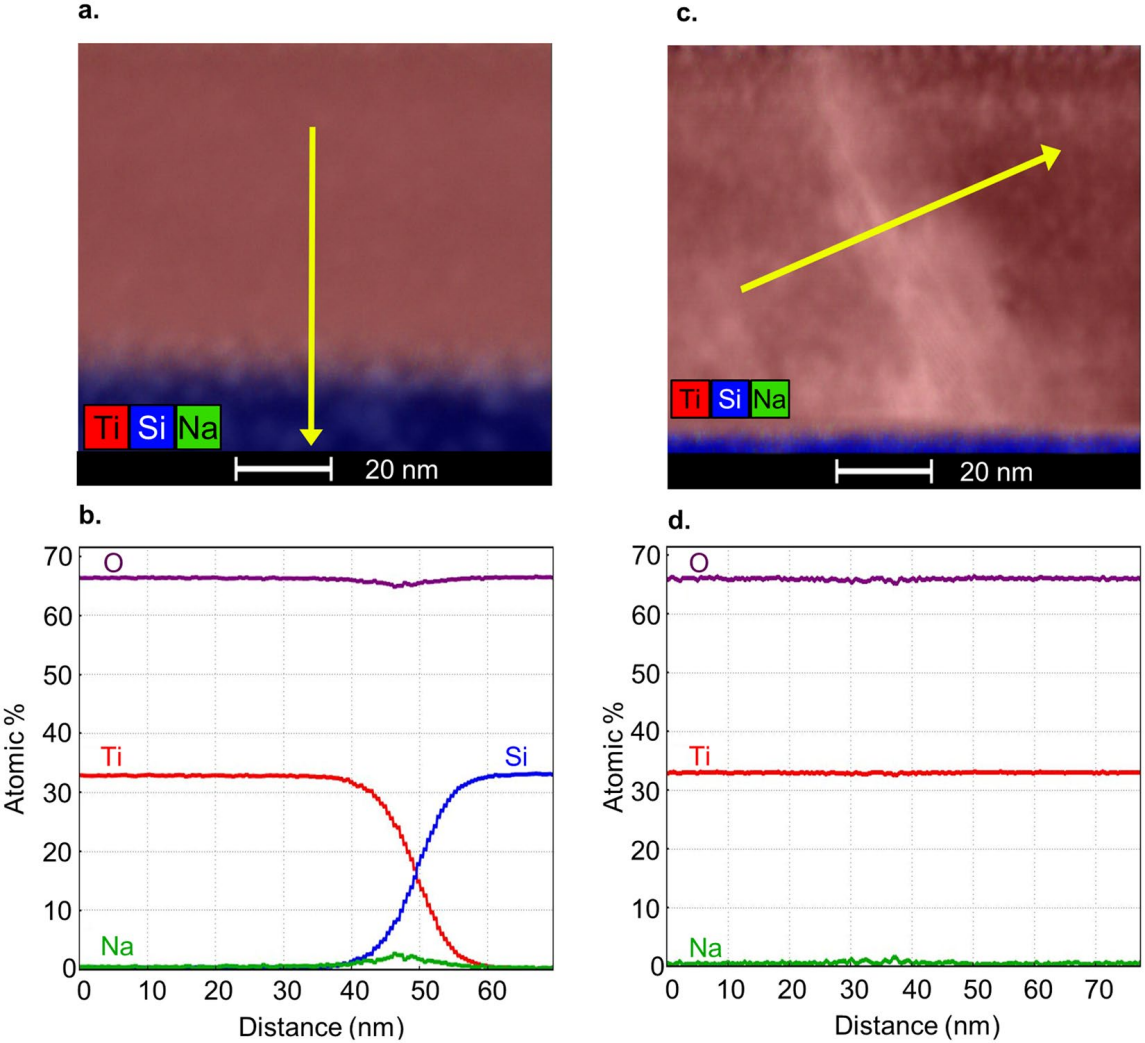
(**a**,**b**) Elemental EDS map and plot trace across the substrate/film interface of the film shown in Fig. 4. (**c**,**d**) Elemental EDS map and plot trace showing an anatase/brookite phase boundary in the film shown in Fig. 6. Na (green) is below the detection limit in the TiO_2_ films and accumulates near the film-substrate interface.

EDS analysis of Ti, O and Na is shown in Fig. 7 of two films grown on EXG and annealed in a manner similar to the film in Fig. 4. The TiO_2_ stoichiometry is confirmed by EDS and there is no change in stoichiometry at or near the grain boundary (<0.35 at%). There is no evidence of Na migration into the film, nor into the grain boundary. There is a clear accumulation of Na in the substrate near the substrate film/interface, however.

### Film thickness and polymorph fraction

**Figure 8:**
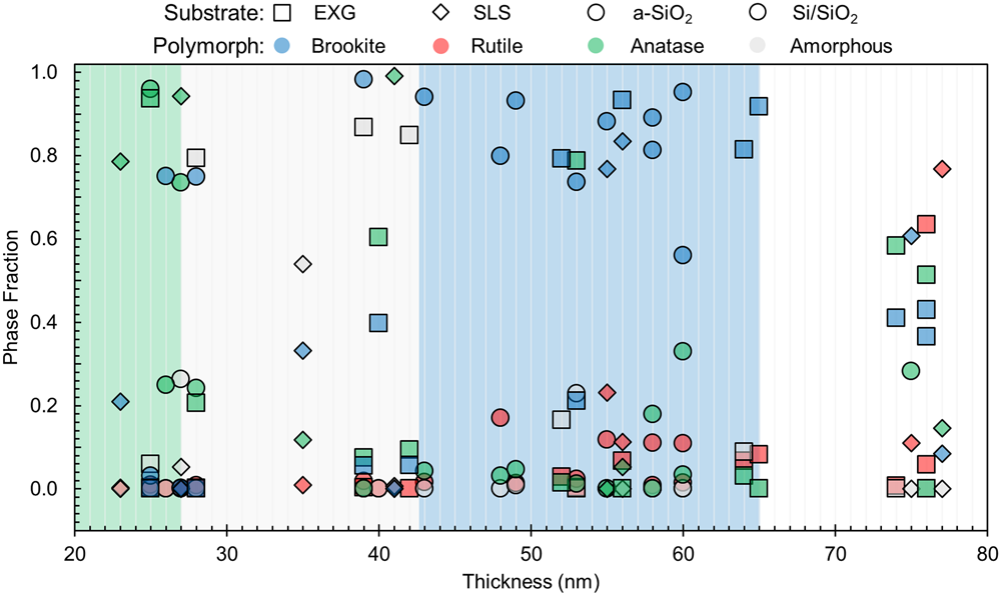
Phase fraction of brookite (blue), rutile (red) and anatase (green) polymorph or amorphous component (gray) in TiO_2_ films as a function of thickness. Na-free a-SiO_2_ and Si/SiO_2_ (circles), low-Na EXG (squares) and high-Na SLS (diamonds) substrates are represented. The uncertainty in thickness is ±5 nm for *d* <50 nm and ±3 nm for *d* >50 nm.

We have reproduced the results described above in many films made on different amorphous substrates that are Na-containing (EXG and SLS) and Na-free (a-SiO_2_ and Si with 100 nm of thermal oxide). Figure 8 shows the results of a phase fraction analysis of a set of samples annealed with the standard protocol described in the methods section using maximum temperature hold times of 0 or 1 minute at 400 °C. (The set includes only films annealed in the same oven to keep the processing variables as similar as possible. In particular, it excludes the film in Fig. 4, where the *in-situ* anneal was used to establish a protocol that would yield brookite.) There is a thickness gradient across most films, which allows the observed phase fraction to be correlated to the thickness of the film in that region. The data in Fig. 8 represent 10 different depositions, with the different classes of substrate denoted by the symbol shape. Each deposition yields several entries on the plot because the film thickness varies across the sample. At each thickness, there are four color-coded points representing the brookite, anatase, rutile and amorphous fractions, averaged over an area approximately 150 µm × 150 µm. One striking feature is that films with brookite as the major phase (above 70% and up to 95%) are clustered in the thickness range of 45 to 65 nm. At the lowest thicknesses, below ~30 nm, anatase is the major phase. Between 30 nm and 45 nm, brookite as the dominant crystalline phase is accompanied by the presence of an amorphous component. This transition corresponds with the size-dependent stabilization order identified by Ranade *et al*.^10^ for TiO_2_ crystallites in solution. Rutile is absent or a minor phase for most of our films; examples of rutile-containing films are given in Figs S2 and S3.

## Discussion

Synthetic routes to brookite TiO_2_ via solution growth suggest that the presence of Na^+^ cations is a driver of the formation of the brookite phase. However, we observe that sodium is not critical to the formation of brookite, on the basis of first-principles calculations, which show that addition of Na into the system does not favor brookite, and because brookite thin film growth occurs independent of the presence or absence of sodium from the substrate. Our analysis suggests it is possible to synthesize brookite TiO_2_ films by PLD on a wide range of substrates, independent of substrate composition and orientation, such as Na-free SiO_2_.

By characterizing the conditions favoring the formation of the brookite phase, we find with this annealing protocol and amorphous films, that brookite preferentially forms in a film thickness range of 45–65 nm, with anatase appearing at smaller film thicknesses. This trend of brookite formation in an intermediate size regime is analogous to the intermediate-size stabilization of brookite in solution growth^10^, although the mechanistic relationship between solid-liquid surface energies relevant to phase selection in nanoparticles, and thickness effects in thin films is not clear. The *in-situ* x-ray study tracking the annealing of the as-deposited amorphous films shows that after an initial crystalline phase forms, few if any transformations occur. This result suggests that phase selection likely occurs during single, independent nucleation events, which can be controlled through annealing rates and final annealing temperatures to possibly achieve phase-pure brookite thin films without the need for assisted growth methods such as epitaxy.

In summary, we synthesize high-phase-fraction (~95%) brookite TiO_2_ thin films by PLD on both sodium-free and sodium-containing amorphous substrates and identify an ideal thickness region for forming the brookite phase with this annealing protocol. We track the formation of the brookite, anatase, and rutile phases via Raman mapping, TEM and *in-situ* XRD and trace their predominance to the processing conditions used, eliminating the previously hypothesized role of Na incorporation as a mechanism for brookite stabilization in thin films. Our results suggest that instead, phase selection in the films is dictated by a single nucleation event, which can be controlled through film processing and structure.

## Methods

### Theory

To model the thermodynamics of possible growth mechanisms we use density functional theory (DFT) as implemented in the Vienna Ab-Initio Simulation Package (VASP)^36^, relying on the SCAN exchange-correlation functional^37^, projector augmented wave method^38^, and a reciprocal space discretization of 25 Å^−1^. We choose the SCAN functional as it was recently demonstrated to generally produce accurate results in phase selection among polymorphs in similar systems^14^, and accurate formation energies for titanium oxides in our own benchmarking.

We evaluate the effect of sodium incorporation into titania polymorphs (i) as accompanied by reduction of Ti, and (ii) as a neutral Na_2_O defect. We enumerate likely phases in the TiO_2_^−^NaTiO_2_ and TiO_2_^−^Na_2_Ti_3_O_7_ spaces respectively, including all known sodiated titania structures (bronze, post spinel, layered, sodium titanate) as well as the common TiO_2_ polymorphs of interest here (rutile, brookite, anatase). In the case of Na^+^ intercalation, we consider both isolated defects and symmetrically distinct orderings on the possible interstitial sublattices in each phase, and obtain the lowest energy configuration across all Na_x_TiO_2_ compositions. To access the Na_2_O^−^defect thermodynamics, we enumerate dilute Na_2_O in TiO_2_ defect configurations, formed by creating a Ti^4+^ vacancy and charge compensating it with 4 Na^+^ ions, over all likely configurations of the sodium ions. In all cases, to correct the well-known problem of reproducing the energy of rutile versus brookite and anatase^39^, brookite and anatase phases are shifted to +0.007 meV/TiO_2_ and +0.023 meV/TiO_2_ with respect to pure rutile respectively, based on experimentally-obtained enthalpies of the three phases^10^. While no similar correction enthalpies are known for the bronze, post spinel, and layered phases, no enthalpy shift of these phases within expected DFT error ranges changes which phase is stabilized in both the Na and Na_2_O cases.

### Film deposition and annealing

Using PLD, we deposit amorphous TiO_2_ films on Corning Eagle XG glass (EXG, MTI corporation), soda-lime silicate microscope slides (SLS, Corning 0215), fused quartz (a^−^SiO_2_, GM associates), and p-type silicon with a 100-nm-thick thermal oxide (MTI corporation). According to the specification sheets, EXG glass has 0.1 wt. % alkali content, SLS has 14% Na_2_O and the fused quartz and p-Si substrates are Na-free. Before deposition, substrates are cleaned with a 1% solution of Liqui-Nox (Cole-Palmer)/deionized water and rinsed in deionized water followed by five-minute ultrasound baths in acetone and then in isopropyl alcohol. Substrates are then dried with compressed nitrogen and stored in a warming oven at 120 °C in ambient atmosphere, until being transferred to the vacuum chamber for deposition.

The films are deposited at room temperature in a vacuum chamber with base pressure near 5 × 10^−7^ Torr, purged with ultra-high purity oxygen for five minutes, and then held at 1.0 mTorr during deposition. Substrates are typically 2 cm × 3 cm and are held stationary at a target-to-substrate distance of 12 cm. This distance produces a thickness variation of about 30% across an individual sample, which is useful to investigate the polymorph fraction as a function of thickness. The ceramic target (99.998% TiO_2_, Materion) is rotated during deposition to obtain uniform ablation. The output energy from the Lambda Physik KrF excimer laser (λ = 248 nm, ~10 ns pulse duration, and 10 Hz repetition rate) is 150 mJ. The laser energy on the target is 60 mJ giving a laser fluence of 0.54 Jcm^−2^. To ensure repeatability, we deposit films in two different chambers at NREL and OSU using similar deposition conditions. The substrates are large enough that they can be cleaved into four sub-samples so that the same amorphous precursor can be processed or analyzed in different ways.

After deposition, the amorphous TiO_2_ films are annealed in one of two ways. For *in-situ* XRD characterization, the anneal occurs in the sample holder in the SSRL beamline according to protocols like that shown in Fig. 4 (0.25 L min^−1^ flowing N_2_ with a heating rate of approximately 60 °C min^−1^ and a cooling rate of approximately 110 °C min^−1^). Otherwise, the anneal occurs in an AET Thermal RX Series Rapid Thermal Processing system under flowing N_2_ with a flow rate of approximately 10 L min^−1^. The peak temperature and processing times are chosen to be near those found at SSRL to produce the brookite and anatase polymorphs. The standard protocol is to ramp to 340 °C at 50 °C min^−1^, then to 400 °C at 20 °C min^−1^, hold at 400 °C for 0 or 1 min, and cool to ambient at 110 °C min^−1^. The films depicted in Fig. 8 have hold times of 0 or 1 minute; the hold time was not a strong influence.

### Optical Spectroscopy

Because the substrate is not rotated during deposition, a thickness variation in the TiO_2_ films is visible as a color gradation in the reflected light. The thickness is quantified using reflection and transmission spectroscopy. Spectra are acquired in the range 250 to 1000 nm using a UV-enhanced xenon light source, a diffraction grating double monochromator and a Si photodetector. With the light beam focused to ~1 mm diameter, reflection (*R*) and transmission (*T*) spectra are acquired in 3–4 different locations across each cleaved sample. The spectra exhibit the intensity modulation characteristic of thin-film interference. *T*, *R* and the interference-free transmission^40^ *T*/(1-*R*) ≈ exp(-α*d*) are fit using established models^41^ with film thickness and film index of refraction as fit parameters, and standard Sellmeier coefficients to give the wavelength-dependent refractive index for the substrate^42^. The evolution of the optical spectra (see Fig. S4) make clear the relative thickness, however, even if the absolute thickness has some error. Optically determined thicknesses are consistent with those measured by cross-sectional TEM on selected samples.

### Raman Spectroscopy

Micro-Raman spectroscopy is performed using a Horiba Jobin-Yvon LabRam800 with 532-nm laser excitation and a beam of about 1 µm in diameter. Data are processed using the LabSpec 5.0 software suite. After background subtraction, peaks are fit with Gaussian functions to identify peak position, amplitude, width, and compared to reference spectra reported in the RRUFF database^33^ for polymorph identification. 2D micro-Raman mapping is performed by inserting a motorized mirror in the laser beam line allowing the laser to be positioned and scanned across the sample, with the detector tuned to the desired wavelength range indicated between the dashed lines in Figs 5b,c, and S4f–h. Optical images are acquired using the Raman system optical microscope, which has 10x, 20x, 50x and 100x objective lenses. Polymorph fraction is calculated from 2D Raman maps or from corresponding 100x optical images over an area of approximately 150 µm × 150 µm.

### X-Ray Diffraction

XRD is performed using the Stanford Synchrotron Radiation Lightsource (SSRL) Beamline 11–3 with an X-ray energy of 12.7 keV. The x-ray beam dimensions are 3 mm by 0.1 mm. Two-dimensional scattering data are collected using a Rayonex charge-coupled device and in a grazing incidence geometry with the x-ray beam held at an incident angle of 3°. Images are calibrated using a LaB_6_ standard and integrated between 10° < χ < 170° (χ is the polar angle) using GSAS-II^43^. The background due to the amorphous substrate is subtracted using the CrystalMaker software package.

### Transmission Electron Microscopy

TEM micrographs are acquired with an FEI Co. Talos F200X transmission electron microscope with scanning capabilities operating at an accelerating voltage of 200 keV. Specimens for TEM are prepared from deposited films via *in-situ* focused ion beam lift-out methods^44^ using an FEI Co. Helios Nanolab 600i SEM/FIB DualBeam workstation. Specimens are ion milled at 2 keV and 77 pA to remove Ga ion beam damage and achieve a final thickness of approximately 80 nm. Structural characterization is conducted by acquiring selected area electron diffraction (SAED) patterns on an FEI Co. Ceta 16 M pixel CMOS camera at a camera length of 410 mm. Platinum from the FIB is used to calibrate the camera constant, allowing SAED reflections to be accurately measured and indexed. Chemical mapping is performed in the TEM using the Super-X energy-dispersive X-ray spectroscopy (EDS) system equipped with four windowless silicon drift detectors, allowing for high count rates and chemical sensitivity to 1 atomic percent.

All data needed to evaluate the conclusions in the paper are present in the paper and the Supplementary Materials. Additional data related to this paper may be requested from the authors.

### Data availability

All datasets are available from the corresponding author upon reasonable request.

## Electronic supplementary material

Supplemental Information
