# Supplementary material for: High-fraction brookite films from amorphous precursors
Source: Sci Rep. 2017 Nov 9;7:15232. doi: 10.1038/s41598-017-15364-y (PMC5680313; doi:10.1038/s41598-017-15364-y)
Supplement: Supplementary file 1 — Supplemental Information [file 41598_2017_15364_MOESM1_ESM.pdf]

Supporting Information for

High-fraction brookite films from amorphous precursors

James E. S. Haggerty[1], Laura T. Schelhas[2], Daniil A. Kitchaev[3], John S. Mangum[4], Lauren M. Garten[5], Wenhao Sun[6,7], Kevin H. Stone[8], John D. Perkins[5], Michael F. Toney[2,8], Gerbrand Ceder[6,7], David S. Ginley[5], Brian P. Gorman[4] & Janet Tate[1]

[1]Department of Physics, Oregon State University, Corvallis, OR 97331, USA.

[2]Applied Energy Programs, SLAC National Accelerator Laboratory, Menlo Park, CA 94025, USA.

[3]Department of Materials Science and Engineering, Massachusetts Institute of Technology, Cambridge, MA 02139, USA.

[4]Department of Metallurgical and Materials Engineering, Colorado School of Mines, Golden, CO 80401, USA.

[5]National Renewable Energy Laboratory, Golden, CO 80401, USA.

[6]Materials Science Division, Lawrence Berkeley National Laboratory, Berkeley, CA 94720, USA.

[7]Department of Materials Science and Engineering, UC Berkeley, Berkeley, CA 94720, USA.

[8]Stanford Synchrotron Radiation Lightsource, SLAC National Accelerator Laboratory, Menlo Park, CA 94025, USA.

1. Texture in TiO_2 films

X-ray diffraction (XRD) is used for phase identification of the different TiO_2 polymorphs as shown in Fig. 3 and 4 in the main text. Such spectra show the XRD signal integrated over the full polar angle χ. Further information about the orientation of the film is evident from examination of the 2D XRD pattern in Fig. S1 where χ is measured along the azimuthal direction. This 2D image shows that brookite is preferentially oriented because the (121) reflection is confined to a small range of χ, while anatase is much less oriented, with its (101) reflection appearing nearly isotropic. We do not know why the brookite is oriented, but the observation suggests that brookite peaks could be missed in conventional XRD geometries that see only the out-of-plane diffraction at χ = 90°.

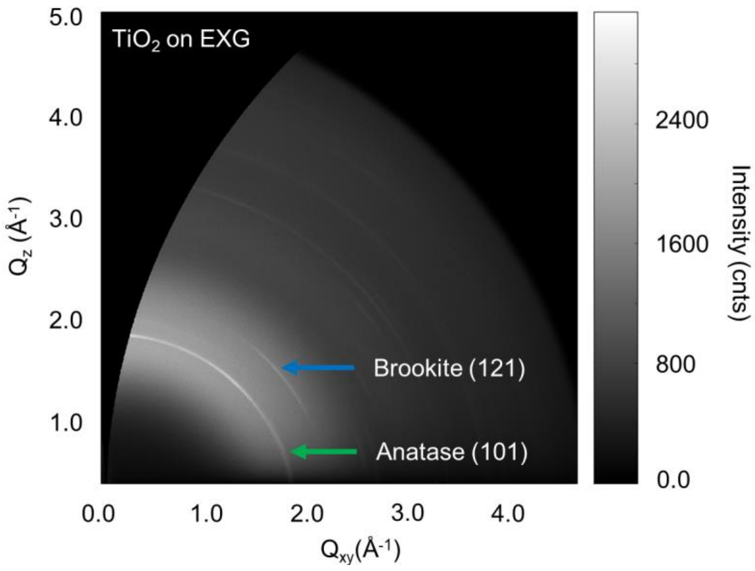

Figure S1. 2D XRD pattern of a 65-nmTiO_2 film on EXG after annealing. The strongest peaks are brookite (121) at Q = 2.166 $Å^{-1}$ and anatase (101) at Q = 1.787 $Å^{-1}$. Brookite (121) has a limited χ range, indicating that brookite crystallites are preferentially oriented.

2. Rutile-containing TiO_2 films

Rutile appears as a minority phase in many of the TiO_2 films described in this paper. In the thickness range *d* < 70 nm, the rutile phase generally appears as circular regions which may be contiguous with any of the other phases. This circular morphology is evident in Fig. S2a and Fig. S2f, which show optical and Raman images of the rutile phase. Fig. S2 shows optical and Raman images similar to Fig. 5 of the main text, but here the substrate is Na-free a-SiO_2 and all three polymorphs are present as well as an amorphous phase. Note also the “veins” in the brookite regions, which are also present in Fig. 5. The veins appear to be brookite of a particular orientation because the Raman spectrum (not shown) has peaks in the same position as the standard non-polarized reference spectrum, but with different intensities. This curious morphology is being investigated further.

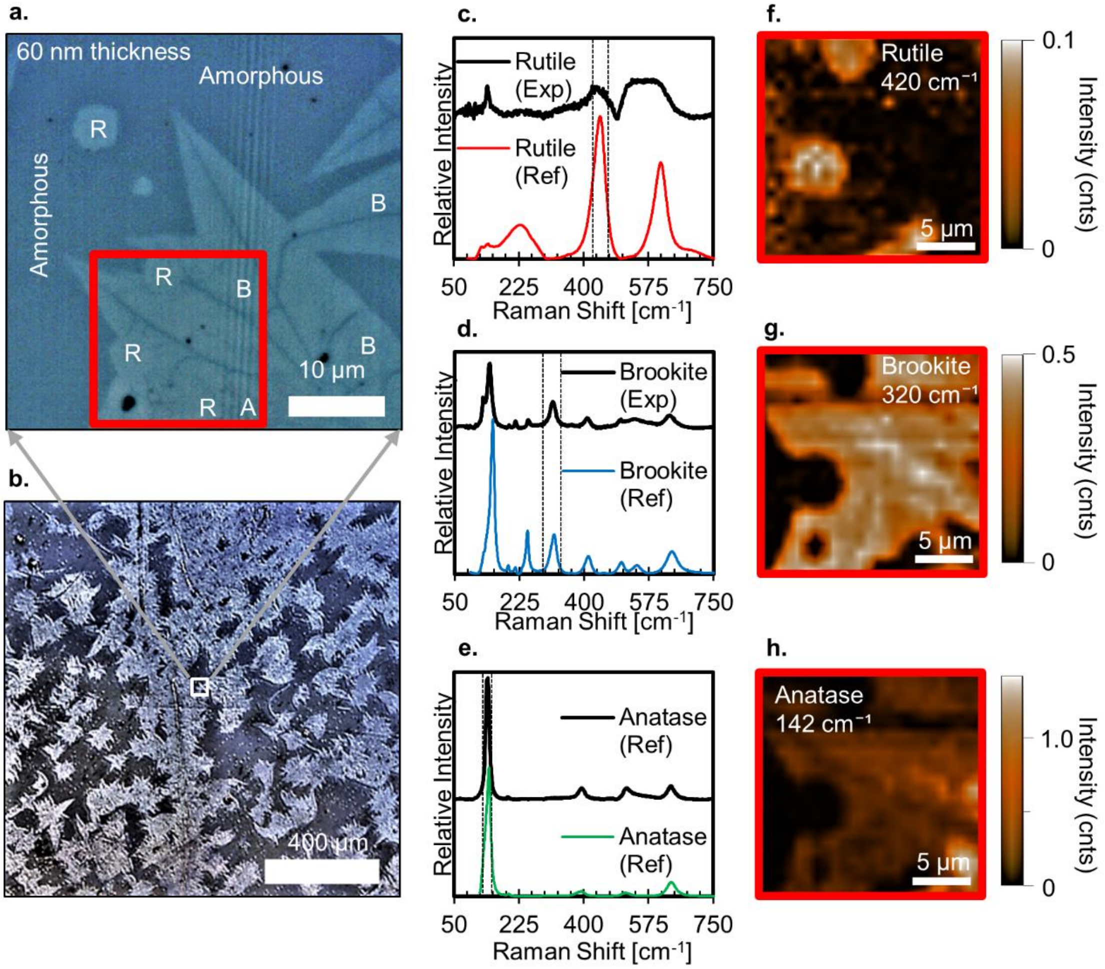

Figure S2. Optical (100x (a) and 20x (b) magnification) and Raman images of a ~60nm TiO_2 film on SiO_2 that contains all 3 crystalline phases and some amorphous phase. (a) High magnification optical image. (b) Low magnification optical dark field image including the entire region in (a). (c), (d), (e) Representative Raman spectra of the regions in (a) labeled R (rutile), B (brookite) and A (anatase). (f), (g), (h) 2D Raman images of the red square in (b) constructed from the wavenumber ranges indicated by the dotted lines in (c), (d), and (e).

Fig. S3 shows optical images of high-fraction brookite films on three different amorphous glass substrates, Na-free SiO_2, low-Na EXG and high-Na XLS. These particular images contain a small amount of rutile, seen as white circles. Although rutile is the lowest-energy polymorph, it forms the least readily from the PLD-depositied amorphous precursors under the anneal conditions described here.

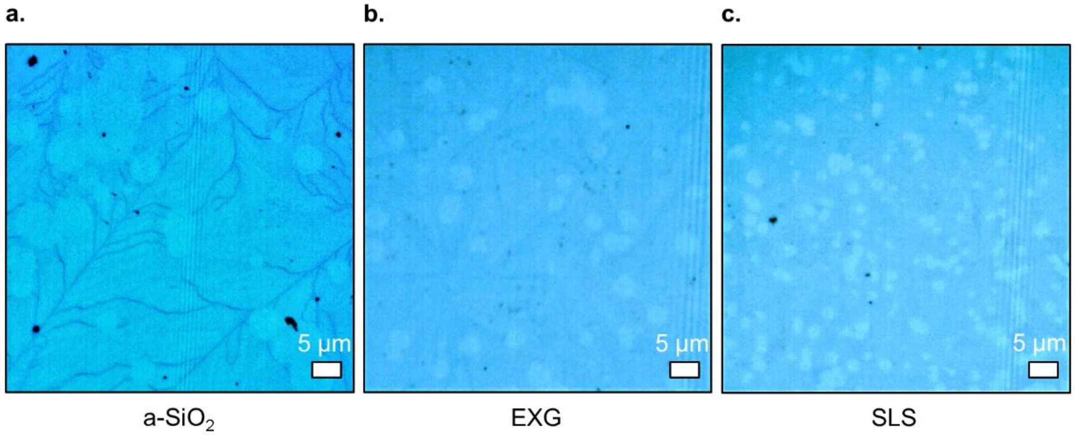

Figure S3. High brookite fraction films of similar thickness on three amorphous substrates (a) Na-free a-SiO_2, (b) low Na-containing EXG, and (c) high Na-containing SLS. The phase composition is identified using micro-Raman spectroscopy and shows only rutile (circles) and brookite.

3. Film thickness and band gap determination

Fig. S4a shows reflection spectra from different parts of two TiO_2 films along a thickness gradient. The thinner sample (green) contains a single constructive interference fringe that shifts significantly with a 5-nm change in thickness. For the thicker sample (blue), he first constructive fringe has moved beyond the range shown and a second, destructive, interference fringe has appeared. The same shift is evident. Fig. S4b shows the transmission and reflection spectra of a 60-nm high-brookite-fraction film on a-SiO_2, along with the reflection-corrected transmission spectrum $T/(1-R) \approx e^{-ad}$. The onset of absorption is at 3.35 eV as determined from the linear region on the Tauc plot in Fig. S4c for the indirect band gap of brookite.

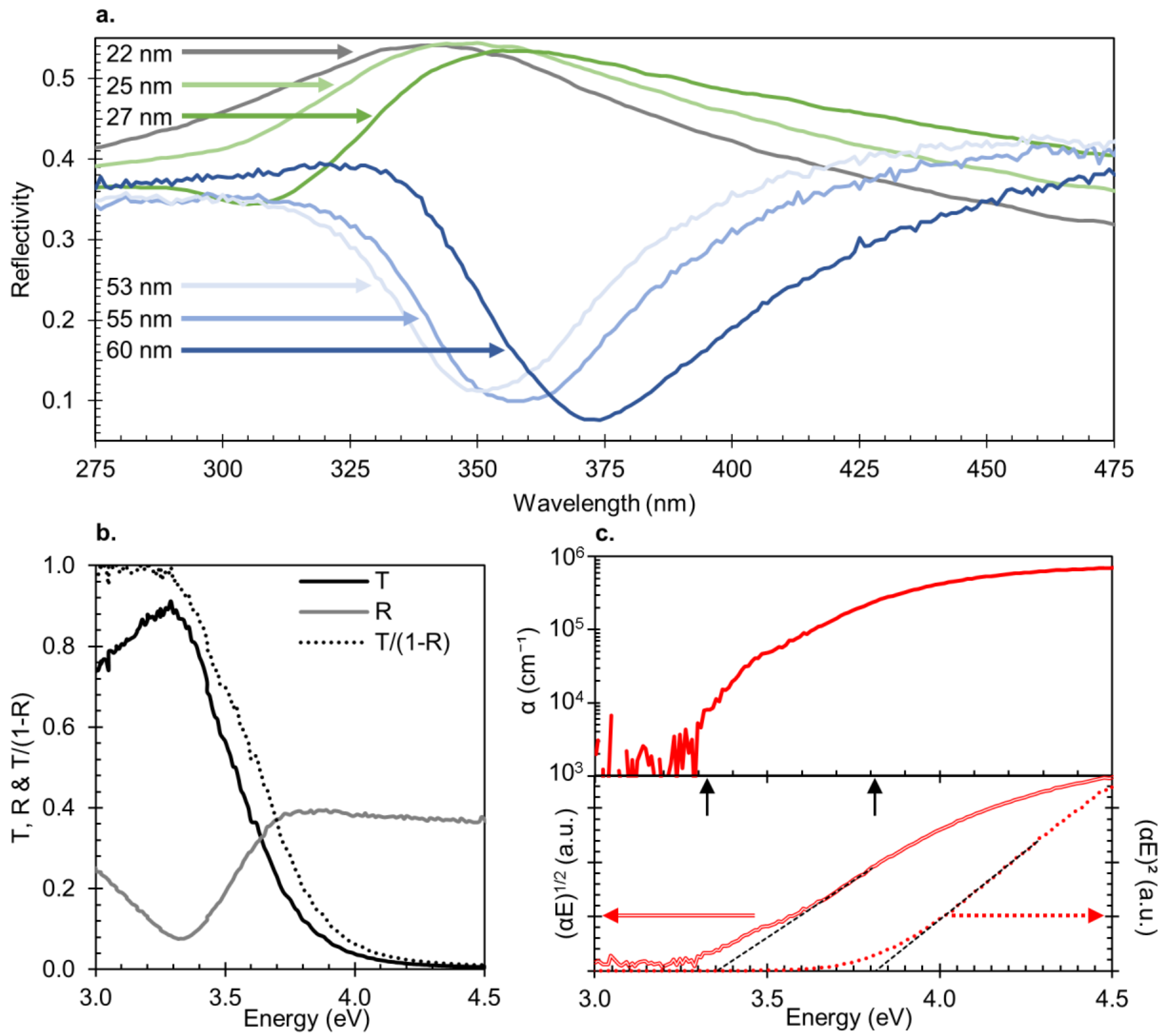

Figure S4. (a) Reflectivity as a function of wavelength showing the shift in interference fringes used to determine film thickness. (b) Transmission, reflection and reflection-corrected transmission of a 60-nm high-brookite-fraction film. (c) Absorption coefficient of the film in (b) with arrows labelling the band gaps obtained if the data are analysed (insets) as indirect (lower value) or direct gap (higher value).
